# Effects of Aging and Distractors on Detection of Redundant Visual Targets and Capacity: Do Older Adults Integrate Visual Targets Differently than Younger Adults?

**DOI:** 10.1371/journal.pone.0113551

**Published:** 2014-12-12

**Authors:** Boaz M. Ben-David, Ami Eidels, Chris Donkin

**Affiliations:** 1 School of Psychology, Interdisciplinary Center (IDC) Herzliya, Herzliya, Israel; 2 Department of Speech-Language Pathology, University of Toronto, Toronto, ON, Canada; 3 Research, Toronto Rehabilitation Institute, Toronto, ON, Canada; 4 Graduate Department of Rehabilitation Sciences, University of Toronto, Toronto, ON, Canada; 5 School of Psychology, University of Newcastle, Callaghan, NSW, Australia; 6 School of Psychology, University of New South Wales, Sydney, NSW, Australia; University of California Davis, United States of America

## Abstract

In the redundant target effect, participants respond faster with two (redundant) targets. We compared the magnitude of this effect in younger and older adults, with and without distractors, in a simple visual-detection task. We employed additional measures that allow non-parametric assessment of performance (Townsend's capacity coefficient) and parametric estimates (Linear Ballistic Accumulator model). Older participants' latencies were slower, especially in the presence of distractors, and their calculated capacity indicators increased with distractors. Parametric estimates indicated that these increases were generated by the older adults' increased difficulty in inhibiting the distractors, and not the results of either improved detection of redundant-targets, or of a generalized slowing of processing.

## Introduction

The detection of a target of interest in a scene is an everyday occurrence. For instance, before crossing the street at night a pedestrian has to look out for the presence of an oncoming vehicle, by detecting its front lights. A visual scene may present distractions (e.g., neon lights) that the onlooker has to ignore, or actively suppress, to behave appropriately. Conversely, a scene can hold multiple sources that indicate the presence of the stimulus of interest (e.g., spotting two front lights rather than one), precipitating a detection response. The latter example sets a cornerstone in the investigation of visual detection and processing. Each front light alone suffices to precipitate the (correct) response. Hence, each target is in fact *redundant* to the other. Evidence shows that the presence of redundant targets can speed up the correct response. The source of this redundancy advantage is the focus of much debate in the literature. It could merely represent statistical facilitation, the product of an independent *horse race* between the two targets [1]. On the other hand, redundancy might have a more profound sensory impact on the processing of visual stimuli, where the two targets are processed in tandem, co-activating the response (e.g. [2]–[4]). It remains an open question whether the (improved) efficiency of processing redundant information is stable across the life span from adulthood to older age. In other words, will older and younger adults benefit to the same extent from redundancy under various conditions and will the processes engendering redundancy gains change with age? In this study we systematically compared younger and older adults in redundant-target situations, using two sophisticated approaches that we outline next.

### The Redundant Target Design

A common way to mimic visual detection in the laboratory is the *redundant target design* (see [5] for a review). In a set of visual stimuli, one is defined as the target (say, the letter X), and the other as the distractor (O). Two items are presented on each trial and the observers respond *Yes* when the display contains at least one target; otherwise they respond *No*. Consequently, a trial in such a design can include two targets (redundant-targets displays, *XX*), one target (single-target displays, *XO* or *OX*), or none (no-target displays, *OO*). Generally, target detection on double-target trials is faster than on single-target trials – the *redundant target effect* (RTE; e.g. [6]). Following Eidels et al. [7] we measure RTE as the advantage in response latency for correct detection of the target in double-target trials over correct detection in the fastest of the single-target trials (or channel, see [2]), rather than to the average of single-targets. For clarification, consider the following hypothetic example, where we test a person who is hard of hearing in an auditory-visual RTE experiment. Latency for visual detection is 400 ms, for auditory detection, 800 ms (as the person is hard of hearing), and for redundant-targets, 400 ms. An RTE calculated with respect to the fastest channel (vision) yields an RTE  =  0, hinting that the participant might have used only the visual information. An RTE calculated with respect to average single-targets yields a positive RTE of 200 ms that is obviously false.

### Age-Related Changes in Visual Redundant Target Detection

How should aging impact redundancy gains in the visual domain? A first attempt to investigate age-related effects on the redundant target design was presented in a series of studies by Allen and his colleagues (e.g. [8]–[10]). Taking these studies together, they point to a significant age-related increase in RTE. To investigate the possible sources of this effect, we discuss next two main accounts presented in the literature on cognitive aging: An age-related change in speed of processing and a change in the efficiency of inhibition of distractors.

#### Speed of processing account

Cognitive slowing, characteristic of the aging process [11], [12], has been taken as a source for deteriorated cognitive performance with aging. In the classic version of the generalized cognitive slowing framework, latencies are a multiplicative function of the task difficulty and of an age-related slowing (see [13]). In that sense, generalized age-related slowing is independent of the task and thus latencies on all tasks should increase by the *same extent* (e.g. [12]),



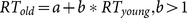
(1)


where RT stands for reaction time, *b* reflects age-related slowdown of central cognitive processes, and *a* is related to peripheral changes. This general equation was found to explain most of the age-related variance across various tasks in several meta-analyses (e.g. [14]; see a recent review [15]). If Equation 1 holds for double-target and single-target trials, an age-related increase in a difference measure like the RTE ensues, namely:



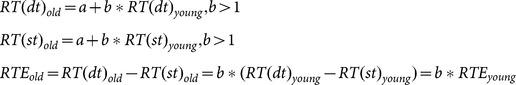
(2)


Where RTE stands for redundant target effect, *st* and *dt* for (fastest) single- and double-target trials, respectively. Therefore, an age-related increase in RTE could merely reflect an age-related generalized slowdown. On the other hand, a *ratio-based RTE* ( =  double-target/fastest single-target) would remain unchanged by age-related slowing (see also [16]). That is,



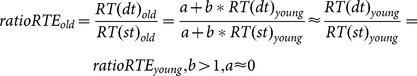
(3)


Alternatively, one may use a log-transform of RT (see [12]) to derive a ratioRTE that is impervious to age-related changes in the speed of processing.



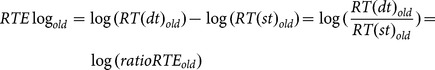
(4)


An examination of the pertinent literature does not provide much support for the speed of processing effect on RTE (the model-fitting analyses we present in the Results section may explain why ratioRTE may have been affected by cognitive slowdown over the lifespan, as the non-decision component of response time was the main source for slowing). In an early examination of ratioRT (using a log version) Allen and his colleagues [8] found age-related effects on RTE to persist after controlling for slowing effects, indicating redundancy gains beyond central slowdown (see also, [16]). More than a decade later, Bucur, Madden and Allen [17], using redundant target design with letter and color as redundant signals, reaffirmed these results. Similarly, in a multimodal audio-visual design [18], an age-related increase in RTE persisted after controlling for age-related slowdown (using a version of a Brinley analysis, following [19]). The above results leave open the possibility that, alongside other sources, speed of processing does play a role in aging effects on the RTE. While some investigators focused on cognitive slowing as the sole cause for increasing RTE with age, others have identified an additional factor, which we discuss next.

#### Inhibition of distractors account

The effects of age on RTE can be linked to the well-established hypothesis on an age-related decrease in the efficiency of inhibitory processes ([20]; for a review, see [21]). With aging, the ability to inhibit the processing of irrelevant information decreases, often inferred as a reduction in selective attention processes. Consider target detection in a distractor-present task: Single-target displays include two signals, one defined as a target (*X*) another as a distractor *(O)*. Thus, the actual displays can be either *XO* or *OX*. Here, an age-related increase in RTE may be generated by a specific slowdown in single-target trials due to difficulties in rejecting the distractor, rather than generalized slowing across trials (alternatively, [22] relate this to increased difficulty in locating a target in single-target-single-distractor displays). Given the central role of Hasher and Zack's [21] inhibition hypothesis in cognitive aging, it is surprising to find that evidence in the current RTE literature is mixed, with distractors found to produce age-related effects [8], reduce age-related effects [9] or have no impact at all [10], as discussed in the next paragraph.

An early study from the Allen lab [8] provides some support for the inhibition of distractors account. Mainly, age-related effects on RTE in a distractor-present task did not persist in a distractor-absent task, where redundant-target trials are compared with single-target-no-distractor trials (e.g., *X alone*). This led the authors to conclude that “part of the redundancy effect is actually a noise reduction effect…older adults had more difficulty in selecting target letters from noise [distractor] letters” ([8] p. 73; the terms used in the current paper were added in brackets). However, other studies have found that older adults' redundancy gains were larger in the *absence* of distractors (Exp. 1 in [9]) or that the effect of distractor presence did not interact at all with age-related effects on RTE [9], [10]. The results of these studies led the authors to reject the inhibition of distractors account and to conclude that “age differences in activation, rather than inhibition, are responsible for age differences in visual search.” ([10] p. 220).

These mixed results clearly call for a fresh examination of the inhibition of distractors account. In our study, we directly compare target detection in distractor-present and distractor-absent tasks for younger- and older-adults. In the distractor-present task, we present *X* as the target and *O* as the distractor, in a two-letter redundant target paradigm. Larger RTEs for older adults in this task may represent superior performance due to redundancy, or a slowdown on single-target trials due to the distractors [23], supporting the inhibition of distractors account. In the distractor-absent task, we present the original double-targets (*XX*), but with no distractors on single-target (i.e., *X* alone) and no-target displays (blank box). Thus, an age-related effect in the distractor-absent task (after controlling for differences in speed of processing) can arise only from an advantage for older adults in accruing information from redundant sources. On the other hand, observing an age-related increase of the RTE in the distractor-present, but *not* in the distractor-absent task, will support the inhibition of distractors account.

To conclude, target detection in double-target trials is generally faster than in single-target trials, the redundant target effect, RTE. This difference is typically larger for older adults. Two main theories that have been presented to explain this age-related effect, generalized cognitive slowdown and inhibition of distractors, but research so far has not unequivocally supported one theory over the other. In the current study we try to shed more light on this question, by examining the general properties of the detection process, and analyzing data at the level of RT distributions, rather than just means.

### Analysis Techniques Based on RT Distribution

Standard calculations of RTE, based on mean RTs as discussed above, provide ambiguous evidence concerning the way people (younger and older adults) process multiple targets. For example, Eidels, Houpt, Altieri, Pei, and Townsend, [24] showed that an RTE can result from a variety of processing systems. Thus, younger and older adults can both exhibit RTE by using completely different processing mechanisms. In the current study we analyze data at both the means (RTE) and the distributional level, with methods developed specifically to solve the type of issues that plague mean response time analyses. The first distribution-based analysis tool is Townsend's capacity coefficient.

#### Townsend's capacity coefficient

Townsend and Nozawa [6] developed the *Systems Factorial Technology* – a set of non-parametric tests for the analysis of RT distributions, helpful in identifying key features of the cognitive processing-system. In particular, they defined a capacity coefficient, C(*t*), that gauges the extent to which target processing in one channel is impaired (C(*t*) <1, limited capacity), left unaffected (C(*t*)  =  1, unlimited capacity), or improved (C(*t*)> 1, super-capacity) by adding a target in the other channel. This index can tell whether (and to what extent) the processing of one target is affected by the presence of another target. Formally, the capacity coefficient is defined as



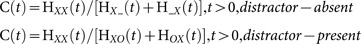
(5)


where H(*t*) is the integrated hazard function. In the S1 Appendix, we provide a detailed description of the measure and how it can be calculated from the distribution of response latencies. Note that C(*t*) coefficients are calculated separately for distractor-absent and -present tasks and then compared against one another.

Redundant targets can facilitate responses. However, redundancy can also impose increased load, which can exceed the capacity resources of older adults. As capacity resources and speed of processing decrease with age (e.g., [14], [25]) older adults may be able to process single targets more efficiently than multiple targets. The capacity coefficient has been proven useful in addressing this issue in general (see [4] for a theoretical review), but had rarely been used in aging research (see a notable exception with audio-visual stimuli in [26]). In the current study, we compared C(*t*) values for older and younger adults. Empirically observing C(*t*)> 1 (super capacity) for older adults will imply an age-related increase in the efficiency of processing multiple targets. On the other hand, C(*t*) <1 (limited capacity; or severely limited capacity, C(*t*) ≤.5) for older adults will imply an age-related decrease in the efficiency of processing as the load of information (i.e., number of to-be-processed items) increases.

#### Testing the different accounts for age-related effects on the RTE with Townsend's C(t)

We can use Townsend's capacity coefficient to discriminate between the generalized slowdown and inhibition of distractors accounts of age-related differences in RTE. First, an age-related generalized slowing of speed of processing predicts that older observers will be slower to detect a target in both single- and double-targets to the same extent. Thus, the numerator and denominator of the C(*t*) index will moderate each other, and their overall C(*t*) plot should not differ substantially from that of younger adults. Similarly, distractor presence may have an impact on the processing of single-target (single-distractor) trials, or even possibly on double-targets (see a discussion on the impact of the stimulus set – both presented and non-pretested items — on detection of double-targets in [5]). However, generalized slowing postulates that these effects should all be guided by the same age-related slowing function and bear no impact on C(*t*).

A different prediction is offered by the inhibition of distractors theory, according to which, age-related effects should be minimal in the distractor-absent task. Since a distractor never appears on the displays, the task does not involve inhibition of distractors. The main difference between age-groups should materialize in the distractor-present task. Specifically, age-related differences will be most evident in single-target-single-distractor trials, as the inhibition of the distractor makes the task a more challenging endeavor for older adults. The predicted difficulties in processing the single-target-single-distractor trials (that require inhibition), versus the relatively preserved processing in double-target trials (that do not require inhibition), will differentially affect the numerator and denominator of the C(*t*) index. As a result, the overall values of this index for older adults will increase in the distractor-present task, as compared to younger adults.

In sum, Townsend's capacity analysis provides an advanced tool that can assist in deciding between the above two accounts of cognitive aging: An age-related increase in the calculated C(*t*) values for the distractor-present task (without a similar increase in the distractor-absent task) supports the inhibition of distractors account, while the lack of such an increase is in accordance with the speed of processing account. And yet, there are age-related differences in several aspects of behavior that influence response times unaccounted for in a non-parametric approach. For example, Ratcliff and colleagues (e.g., [27]) have repeatedly shown that older participants tend to be slower than younger participants because they are more cautious when responding and because they are slower in non-decision aspects of observed response times (such as sensory factors and the time taken to execute the motor response once a decision is made). In the current study, we seek confirmation of Townsend's coefficient by employing a complementary technique of calculating workload capacity developed by Eidels, Donkin, Brown, and Heathcote [28] that explicitly models these differences between younger and older participants.

#### Linear Ballistic Accumulator (LBA) model - A parametric approach to capacity

Our model-based method for calculating capacity is based on an evidence accumulation model of response times arising from simple decisions, the Linear Ballistic Accumulator (LBA; [29]). In evidence accumulation models, a decision is assumed to involve the collection of information relevant for the current task that is then accumulated as evidence for potential responses. A decision is made once enough evidence has been collected for one of the particular responses. The LBA model was developed as a simpler alternative to the Ratcliff diffusion model [30]. The diffusion model has been used to demonstrate the influence of aging on a multitude of simple cognitive tasks (e.g. [31]-[34]), such as letter discrimination and lexical decision. The LBA is similar to the diffusion model in the sense that it can be used to re-describe choice and response time distribution data as a set of psychologically meaningful latent variables, such as response caution and the rate of evidence accumulation.

A typical decision on a single trial in the LBA model proceeds as follows: Each response begins with a random amount of evidence sampled from between 0 and *A*, and accumulates at a linear and fixed (i.e., ballistic, or without noise) rate that is sampled from a normal distribution with mean, *v*, and standard deviation, *s*. Evidence accumulates until a threshold amount, *b*, is collected and a response is emitted. The observed response time is the sum of the time taken for evidence to reach threshold plus the time taken for non-decision aspects of response time, *t_0_*, such as stimulus encoding and the execution of the motor response.

The current study will focus on three parameters: The threshold amount of evidence for a response, non-decision time and the average rate of evidence accumulation. The response threshold, *b*, is often associated with response caution, since higher thresholds reflect the need for more evidence in order to make a decision. Non-decision time, *t_0_*, is related to sensory and motor factors. Specifically, in our paradigm it can represent visual-sensory factors and hand movement factors that generally deteriorate in aging (e.g., see [35]). Both parameters, response threshold and non-decision time, have been found to be larger for older than for younger participants, reflecting greater caution when responding and slower encoding and motor action time, respectively [31]–[34]. The rate of evidence accumulation, *v*, is going to be our index of performance. Evidence accumulation rate is the speed at which appropriate information is collected from a display. So, for example, in target trials, an efficient observer, or a strong signal, will lead to faster collection of evidence regarding the presence of a target, and consequently more accurate and faster responses. It is generally assumed that accumulation rate reflects the cognitive speed of processing (e.g., [27]).

Eidels et al. [28] extended the standard LBA model to explain behavior in redundant target design experiments. They assumed that separate channels existed for processing the presence of each of the targets. In our experiment, this amounts to assuming that observers process evidence for and against the presence of a target in each of the two on-screen locations, where the target letter might appear. Therefore, the model assumes a race between four LBA accumulators, two at each location, one collecting positive evidence and the other negative evidence. A *target present* response is elicited when a target is detected in either location, and a *no target* response is elicited when the absence of a target is detected in both locations.

The model is parameterized in such a way as to yield a parametric equivalent of Townsend's capacity coefficient. The rate at which evidence for the presence of a target is accumulated is estimated separately for redundant-target (*v_RT_*) and single-target (*v_ST_*) trials. The ratio between the accumulation rates in the two types of trials gives a measure of the capacity of the system, that can be read similarly to Townsend's C(*t*). Capacity is said to be super if evidence accumulates more quickly in each accumulator when there are two targets present, rather than one (*v_RT_/v_ST_*> 1). Capacity is said to be limited, if the evidence for each target accumulates more slowly when there are two targets (*v_RT_/v_ST_* <1). Finally, if the accumulation rate for target(s) is equivalent in double- and single- target trials (*v_RT_/v_ST_*  =  1), then capacity is said to be unlimited.

In sum, the LBA model provides a parametric approach to capacity that allows for a separate examination of different aspects of latency distribution that can be affected by aging. Mainly, the rate of evidence accumulation, *v*, represents changes in generalized speed of processing, and non-decision time, *t_0_*, represents sensory and motor differences. The LBA analysis thus presents a novel powerful tool that sheds further light on the two rivaling accounts for age-related increase in RTE for older adults. A generalized decrease in accumulation rate for older adults, across the different types of trials and tasks, would support the speed of processing account. In contrast, a specific age-related decrease in accumulation rate for single-target-single-distractor trials, that is larger than any other trials, will support the inhibition of distractors account.

### The Present Study

The purpose of the present study is to test the impact of age and distractors on the processing of redundancy in visual stimuli. We conducted a prototypical redundant-target experiment, testing both younger and older adults in two types of tasks: Distractor-present, where targets (*X*) and distractors (*O*) could be presented, and distractor-absent, where the stimulus set consisted only of the target letter. In our analysis, we employ standard and log-transformed RTE calculations, based on average latencies, as well as analyses of capacity based on the distribution of response latencies, C(*t*) and estimation of LBA parameters.

Two separate (possibly opposing) accounts for age-related changes are being compared, inhibition of distractors [11], [12] and speed of processing [20], [21]. Namely, after controlling for speed of processing, a lack of an age-related difference in RTE and workload-capacity will support the speed of processing account. In contrast, an age-related increase on tested measures (RTE, C(*t*) and LBA) in the distractor-present task, after controlling for speed of processing, will support the inhibition of distractors account. As both possible accounts have robust support in the literature, we employ an arsenal of tools to delineate between them. If all tests converge on the same conclusion, it will go a long way in choosing between the two. In the conclusion of this study, we also examine the possible impact of age-related sensory degradation on our results.

Our main hypothesis, based on the literature described above, is an interaction of age-group (young, old) X task (distractor- absent and -present) on all tested measures (RTE, C(*t*) and LBA). From a theoretical perspective, the hindering effect of distractors on processing efficiency would be greater for older adults as they are less capable of suppressing these irrelevant sources of information – inhibition of distractors account. We do not commit to this prediction, but rather employ critical tests for its evaluation.

We have a set of specific predictions to test, depending on the measure used. With respect to RTE: (1) We expect faster performance with two targets presented as opposed to just one, documenting redundancy gains. (2) We expect redundancy gains to be greater in the distractor-present task, for both age-groups. The presence of distractors on single-target-single-distractor displays should slow down performance, whereas double-target displays present no distractors. (3) Most importantly, the inhibition of distractors theory predicts that the increase in RTE in the distractor-present task would be greater for older adults, as they are less capable of suppressing the distractors in single-target-single-distractor trials. (4) Finally, a log-RTE is employed to control for speed of processing. If the age-group X task interaction stands – it will support the inhibition of distractors account.

A similar set of specific hypothesis are tested with respect to work-load capacity. (1) We expect capacity estimates to increase in the distractor-present task, for both age-groups. Since capacity is a relative measure, it can increase either because the efficiency of processing multiple targets is improved, or because the efficiency of processing single-target-single-distractor displays is hampered by the presence of the distractor. (2) We assume the latter hypothesis, in accordance with the inhibition of distractors account. As a result, we expect the increase in capacity in the presence of distractors to be larger for older adults, as they have more difficulties in ignoring the irrelevant distractor presented.

The LBA parametric approach to capacity allows a fine-grained analysis of capacity, and generates a detailed set of hypothesis. (1) As the LBA capacity estimate controls for speed of processing differences between older and younger adults, a replication of the age X task interaction supports the inhibition of distractors account. (2) In separate tests of the evidence accumulation rate parameter, we expect to see a specific age-related effect for distractors in single-target-single-distractor trials. (3) Importantly, the speed of processing account predicts a main effect for age-group on accumulation rates. Alternatively, if accumulation rates are not the source for age-related effects, one should expect a main effect for age on the non-decision time parameter, indicating a motoric or sensory source for age-related effects.

## Method

### Participants

22 younger adults (*M*  =  22.3 years, *SD*  =  4.9) and 22 older adults (*M*  =  71.9 years, *SD*  =  3.2) participated in this study. The younger adults were undergraduates at the University of Toronto Mississauga, and the older adults were volunteers from the local community. All older participants were enrolled in the participant pool of the Human Communication Lab, University of Toronto Mississauga. They were assessed once a year and found to have basic cognitive and sensory scores within the normal range for their age-group. Participants were paid 10$/hr. By self-report, all participants enjoyed good ocular health. All participants had a minimum Snellen fraction within clinically normal limits (20/20 and 25/20 for younger- and older-adults, respectively) in the left eye, right eye, or both (averages for corrected binocular near vision: 13.0/20, *SD* = 1.3/20 and 19.8/20, *SD* = 5.3/20, for younger- and older-adults, respectively). To further assess basic cognitive abilities, all participants completed the Mill Hill IQ Vocabulary subtest [36], and achieved a minimum score of 9/20, corresponding to normal scores for native English speakers. The average Mill Hill score was 14.3/20 (*SD* = 2.4/20) for younger adults and 15.4/20 (*SD* =  2.3/20) for older adults, *t*(42)  =  1.5, *p*  = .07, characteristic of these populations (e.g., see [37], [38]).

### Ethics Statement

This study had approval from the Research Ethics Board of the University of Toronto, and all participants provided their written informed consent. All participants were adults and signed the written consent form.

### Stimuli, Apparatus and Design

In the distractor-absent task, the letter X was used as the target, whereas in the distractor-present task, the letter X was the target and the letter O was the distractor. All letters were presented in Arial bold, font size 90, which at a viewing distance of 60 cm amounted to 2.43 degrees of visual angle. On a trial, a white rectangular frame (4.25” X 3.19”) was presented at the center of the screen, in which a letter could appear above or below the center of the frame. In the distractor-present task, 25% of the trials were no-target trials (*O* displayed at the top and bottom positions), 50% were single-target-single-distractor trials (*X* above *O*, or *O* above *X*), and 25% were double-target trials with *X* in both positions. In the distractor-absent task, on 25% of the trials the frame was blank (no-target trials), in 50% the letter X appeared within the frame either at the top or bottom position (single-target-no-distractor trials) and in 25% of the trials, the letter X appeared in both the top and bottom positions (double-target trials). The stimuli were displayed white on a black background.

The participants were instructed to press one key (*Yes*) if at least one of the letters in the display was the target (X) and another key (*No*) if the target was not present. Trials were response terminated. The study consisted of two experimental sessions, each about an hour long, separated by 2-7 days. Each session consisted of two tasks: Distractor-present and distractor-absent. The order of these tasks was fully counterbalanced. Each task consisted of ten blocks, five in each session, with 160 trials each (1600 trials per participant, per task).

### LBA Model Description

The LBA model that we fit had 15 free parameters (model selection using Bayesian Information Criterion indicated that this model gave the most parsimonious account of the data). First, start point variability (*A*), response threshold (*b*) and non-decision time (*t_0_*) parameters were estimated separately for distractor-present and distractor-absent tasks (denoted by the subscripts DP and DA, respectively), but were fixed across no-, single- and double-target trials. We also estimated separate response thresholds for target present and absent responses, since it is possible that participants might collect different amounts of evidence to affirm the presence of the target (respond *Yes*, denoted by the subscript *Y*) than to reject in its absence (respond *No*, denoted by *N*). This set of assumptions yielded four *b* parameters (*b_Y-DA_*, *b_Y-DP_*, *b_N-DA_*, *b_N-DP_*), two *A* parameters (*A_DA_*, *A_DP_*), and two *t_0_* parameters (*t_0-DA_*, *t_0-DP_*) values. The standard deviation of the between-trial distribution of drift rates, *s*, was set at 0.25 to satisfy the scaling property of response time models. The remaining free parameters were accumulation rate parameters.

The parameterization for evidence accumulation rates followed closely Eidels et al., [28]. For correct target-detection responses, there were four accumulation-rate parameters: Two for trial-type (redundant-target and single-target, denoted by RT and ST, respectively) and two for task-type (distractor-present and -absent, denoted by DP and DA, respectively). This combination resulted in four relevant accumulation rates — *v_RT,DP_*_;_ *v_RT,DA_*_;_ *v_ST,DP_*_;_ *v_ST,DA_*. For incorrect target detection in no-target trials, there was one accumulation rate, *v_NT_*. There were also two rate parameters for target rejection (*no target* responses): A rate parameter for miss response, when there was a target present in the display (*v_∼T_*), and for a correct rejection, when there was no target present (*v_∼NT_*). These seven rate parameters can be combined to encompass the full range of possible combinations of stimuli (see Eidels et al.'s Table 1 for the full details [28]).

**Table 1:** A summary of mean response latencies (in ms).

	Younger adults		Older adults	
	Distractor-absent	Distractor-present	Distractor-absent	Distractor-present
Redundant-target	390 (51)	406 (58)	445 (54)	454 (50)
Fastest single-target	396 (52)	419 (55)	451 (57)	480 (52)
No-target	491 (63)	505 (71)	577 (103)	594 (100)
Average RT	422 (53)	442 (59)	484 (57)	507 (55)
RTE	6***	13***	6***	26***
RTE log-transform	0.006***	0.015***	0.005***	0.024***

*** *p* <0.001, RTE  =  RT(faster single-target) – RT(double-target). *Note*: Fastest single-target was calculated individually for each participant and then averaged across all participants in the task and age-group; Average RT is the average of all trials across participants in the task and age-group. Standard deviations are presented in parentheses.

Parameters for the model were estimated separately for each of the 44 participants by maximizing the joint likelihood of each individual's complete set of response times and choices (for details on the likelihood calculations, see [28], [29]). We used the SIMPLEX search algorithm to obtain the best fitting parameters. We note that the LBA model can sometimes give unreliable parameter estimates when accuracy is at ceiling. However, this occurs because it is difficult to estimate both threshold and rate parameters separately (because they trade off against one another). Thankfully, here the thresholds are held constant across a number of conditions, such that there are enough errors to constrain them. As such, it is possible to get stable estimates of rate parameters.

## Results

### Accuracy

Overall accuracy was high. Accuracy data was submitted to a mixed 2 (between-participants – age-group: older, younger) X 2 (within-participants – task-type: distractor-present or -absent) X 4 (within-participants – trial-type: double-targets, single-target on top, single-target at the bottom, no-target) ANOVA. Accuracy varied slightly across trial-types with rates of 99.8%, 99.7%, 99.1%, and 96.9% for double-, two single- (a single target on top, at the bottom), and no-target trials, respectively (main effect of display-type, *F*(3,129)  =  73.88, *p* <.001). A main effect of age indicated that accuracy was slightly higher for older adults (99.1% vs. 98.6%, *F*(1,42)  =  6.8, *p*  = .01). No main effect was found for the type of task (*F*  =  1.0).

### Mean RTs

Analyses of RT are restricted to correct responses. Responses faster than 200 ms and slower than 1,200 or 900 ms, for older and younger adults, respectively, were discarded (1.45% of trials). Mean RTs are presented in Table 1 for younger (leftmost columns) and older adults (rightmost columns). RTEs are computed conservatively for each participant by comparing double-target RTs against the RT of their fastest single-target trial. For the complete individual data, please refer to S1 Table.

**Figure 1:**
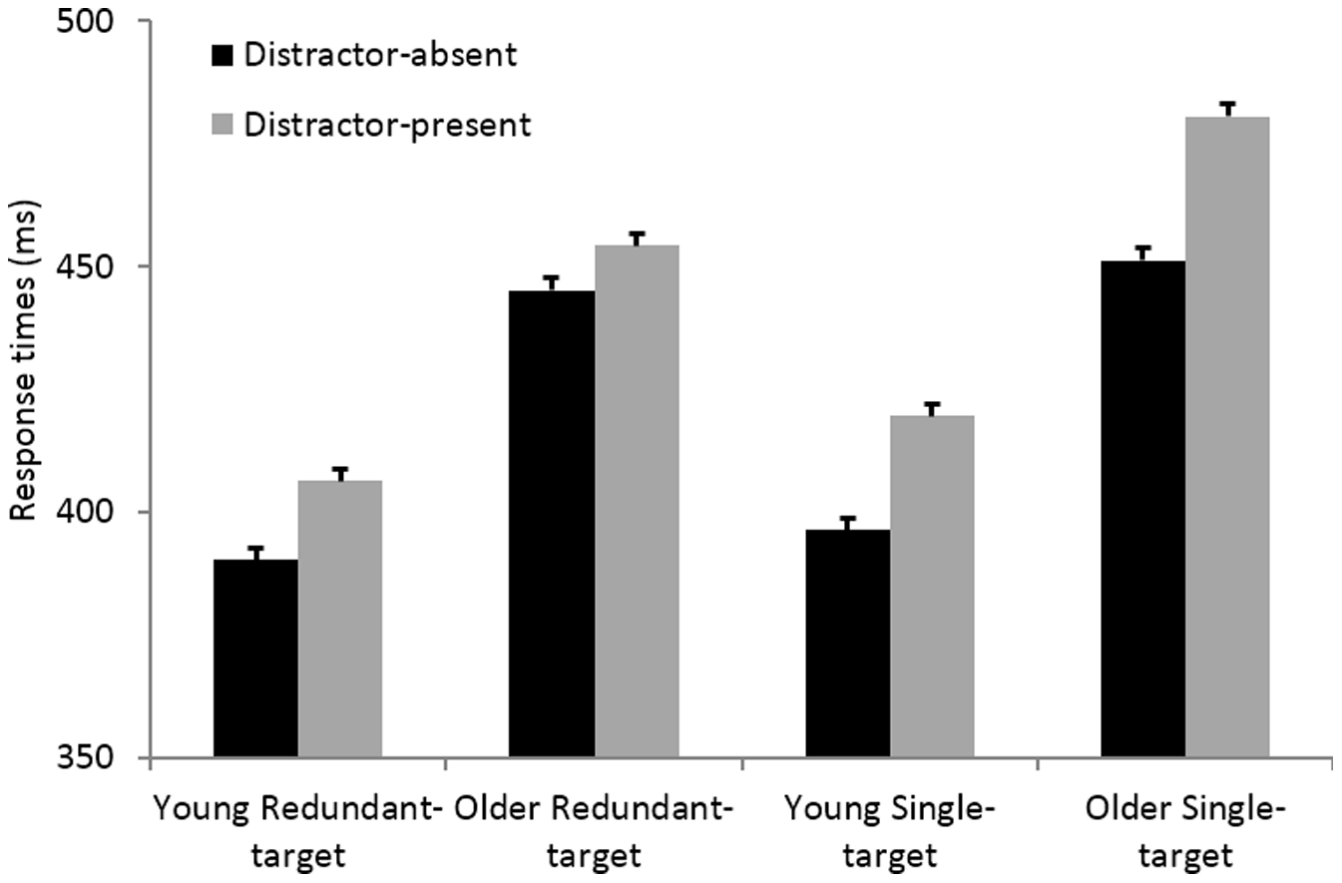
Response times for older and younger adults, across the two tasks (distractor-present and distractor-absent) and two types of target trials (single- and redundant-target).

Table 1 reveals the overall trend: Older adults are generally slower than younger adults (by an average of 62 ms), yet in the distractor-absent task, RTEs do not differ between age-groups (6 ms in both). Turning to distractor-present task, RTEs for older adults are twice as large as RTEs for younger adults. To examine this statistically, latencies were submitted to 2 X 2 X 2 repeated-measures ANOVAs with task-type (distractor-present vs. –absent) and redundancy (fastest single-target vs. redundant-target trials) as within-participant factors, and age-group (younger vs. older adults) as a between-participant factor. The analysis revealed a main effect for redundancy, *F*(1,42)  =  153.3, *p* <.001, *η_p_^2^*  = .79, indicating a significant RTE across conditions and age-groups; a main effect for task-type, *F*(1,42)  =  12.4, *p*  = .001, *η_p_^2^*  = .23, reflecting slower responses in the distractor-present task (note, the main effect describes an overall slowdown, whereas both tasks present the same double-target trials); and a significant main effect for age-group, *F*(1,42)  =  12.9, *p* <.001, *η_p_^2^*  = .24, indicating that older adults were overall slower to respond. The analysis also revealed a significant interaction of task-type and redundancy, *F*(1,42)  =  64.5, *p* <.001, *η_p_^2^*  = .61, reflecting overall larger RTEs in the distractor-present condition; a significant interaction of age-group and RTE, *F*(1,42)  =  10.0, *p*  = .003, *η_p_^2^*  = .19, indicating that older adults had larger RTEs; and a significant interaction of the three variables, task-type, age and redundancy, *F*(1,42)  =  14.8, *p* <0.001, *η_p_^2^*  = .26. A visual presentation of the triple interaction is available in Fig. 1.

Next, to explicate the nature of the triple interaction, we conducted post-hoc ANOVAs of redundancy (fastest single-target vs. double-target) and age-group (younger vs. older adults) separately for distractor-present and distractor-absent tasks (Bonferroni corrected for the 18 possible comparisons among means). First, in the distractor-absent task, we found significant main effects for redundancy, *F*(1,42)  =  35.8, *p* <.001, *η_p_^2^*  = .46, and age-group, *F*(1,42)  =  11.5, *p*  = .001, *η_p_^2^*  = .26, that did not interact (*F* <.1). Thus, even though older adults were slower to respond, no age-related effects were observed for the RTEs, as both younger and older adults exhibited RTEs of the same extent. Second, in the distractor-present task, we found significant main effects for redundancy, *F*(1,42)  =  151.8, *p* <.001, *η_p_^2^*  = .78, and age-group, *F*(1,42)  =  11.4, *p*  = .002, *η_p_^2^*  = .21, and, more importantly, a significant interaction of the two, *F*(1,42)  =  16.8, *p* <.001, *η_p_^2^*  = .29. Here, not only were older adults overall slower, their RTEs were larger than for younger adults (26 vs. 13 ms). In sum, the triple interaction is the consequence of an age-related increase in RTE in the distractor-present task, which vanished in the distractor-absent task.

#### Analysis of speed of processing on latencies

An age-related increase in RTE can be the simple outcome of age-related generalized cognitive slowing. Indeed, older adults were overall slower than younger adults to respond. Given the latency analysis presented above, the speed of processing account may already seem unlikely. Age-related overall slowing was observed in both distractor-absent (62 ms) and distractor-present (65 ms) tasks to a similar extent (in the omnibus ANOVA discussed above, no significant interaction of age-group and task-type was observed, *F* <.1), yet an age-related increase in RTE was observed only in the distractor-present task. Nevertheless, the support for the speed of processing account in the literature prompted us to examine whether controlling for age-related changes in speed of processing can eliminate the age-related RTE differences.

To partially control for age related slowing, we repeated the analysis with the log-transform of the data, following a similar analysis by Allen et al. [8], as presented in the bottom rows of Table 1. The log-transform analysis replicated the main results observed in the analysis of raw latencies, with a triple interaction of task-type, redundancy and age, *F*(1,42)  =  10.4, *p*  = .002, *η_p_^2^*  = .20, reflecting an age-related effect for log-transformed RTE in the distractor-present task, *F*(1,42)  =  9.4, *p*  = .003, *η_p_^2^*  = .18 (approaching significance, after a Bonferroni correction for multiple comparisons), that was not replicated in the distractor-absent task, *F* <1. In sum, after controlling for speed of processing, age-related effects on RTE in the distractor-present task were still significant (even if somewhat diminished). Age-related changes in speed of processing do not appear to be the main cause for age-related effects on RTE.

### Townsend's Capacity Coefficients

**Figure 2:**
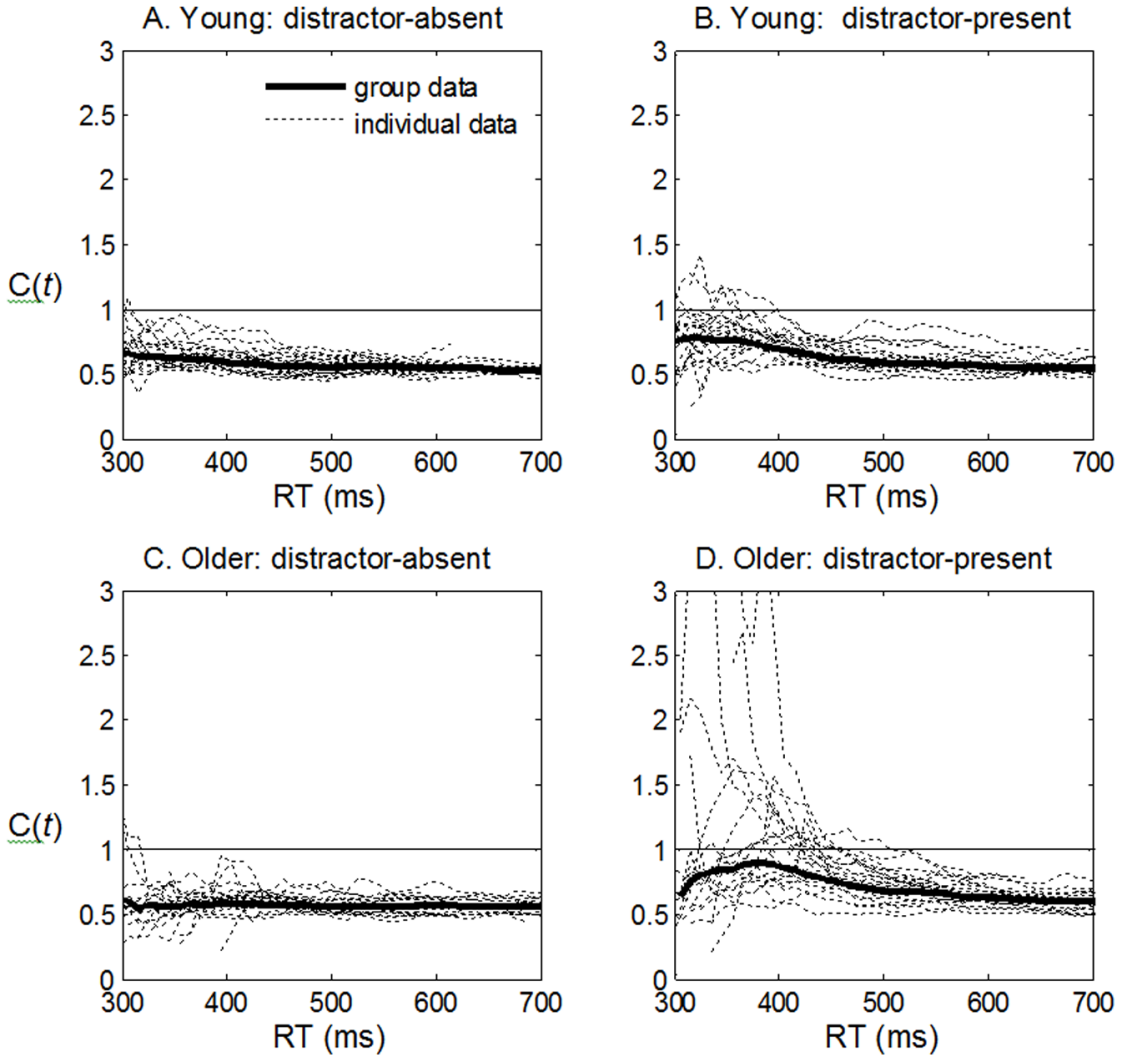
Capacity coefficient for younger and older participants in distractor-present and distractor-absent tasks. The thin dotted lines give C(*t*) estimates of each individual member of the pertinent group, and the thick solid line gives the group values, aggregated across all group members. C(*t*)  =  1 is the threshold for unlimited capacity – processing of one channel is unaffected by the presence of another target; C(*t*)> 1, super-capacity, implies that redundant-targets lead to superior performance; C(*t*) <1, limited capacity, implies that target processing in one channel is impaired by adding a target in the other channel.

The analysis of C(*t*) values for older and younger adults reinforces the analysis of average latency. Mainly, age-groups differ on capacity coefficients only in the distractor-present task. Fig. 2 shows C(*t*) values for younger (panels A and B) and older adults (panels C and D), separated by task (distractor -present or -absent), calculated individually (dotted line), then aggregated across all group members (solid line). In the distractor-absent task, C(*t*) values for both groups (Fig. 2, panels A and C) were similar, and almost completely below unity, implying limited capacity. In the distractor-present task, for more than half of the older adults, many C(*t*) values exhibit super-capacity at some *t*, whereas C(*t*) for younger adults rarely exceeds unity (Fig. 2, panels B and D). In other words, in the distractor-present task, RTEs for older adults reflect improved processing of redundant-targets *relative* to single-target-single-distractor trials. This does not necessarily mean that older adults are better in detecting double-targets, but that perhaps they were worse in single-target-single-distractor trials.

**Figure 3:**
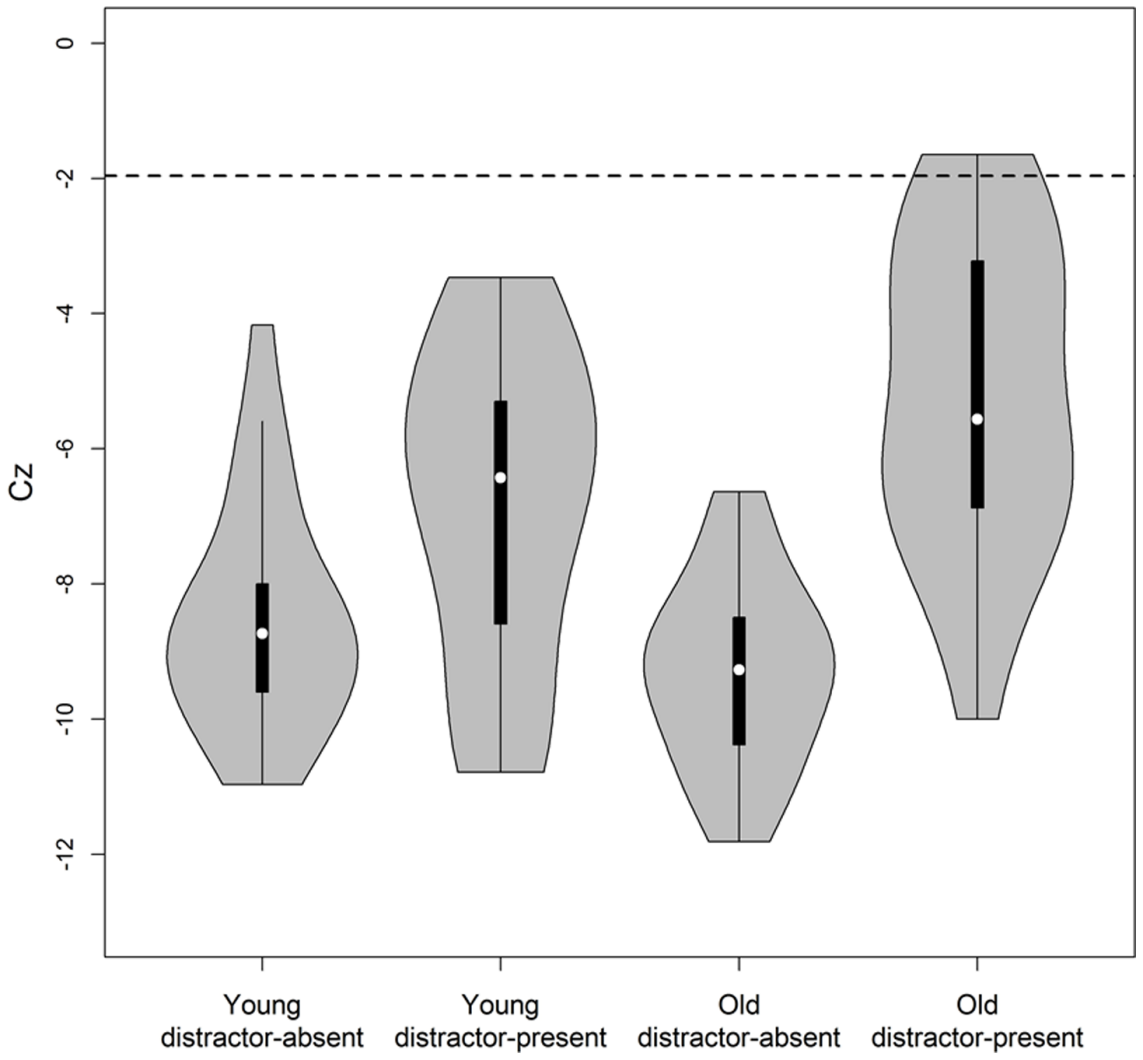
The distribution of Houpt and Townsend's [39] capacity test statistics, Cz, for younger and older adults across the two tasks (distractor-present and distractor-absent). Distributions are plotted using violin plots. These plots consist of a box plot in the center of each violin, with a white circle representing the median, a black rectangle outlining the central 50% of the distribution, and a solid line extending to two standard deviations from the median. The grey area of the violin is a smoothed plot of the distribution of Cz values using a kernel density estimator. The dotted line represents the cut-off for statistically significant limited capacity.

Following the visual inspection of Fig. 2, we use the statistical tool for testing capacity recently offered by Houpt and Townsend [39]. Capacity coefficients are summarized using a statistic, C*_Z_*, that follows a standard normal distribution. The technique yields a single value per participant per condition. Each individual's capacity value can be judged as reliably limited if C*_Z_* is less than -1.96, and can also be compared across conditions. Fig. 3 plots the distribution of C*_Z_* values from each of the four conditions in our experiment. An inspection of Fig. 3 finds limited capacity for all individuals in all conditions (but for two older adults who had only marginally significant limited capacity in the distractor-present condition).

The pattern of C_Z_ values reinforces the visual inspection of the C(*t*) functions in Fig. 2. We submitted the C_Z_ values to a 2 X 2 mixed design repeated measures ANOVA with task-type (distractor-present vs. –absent) as a within-participant factor, and age-group (younger vs. older adults) as a between-participant factor. The analysis revealed a main effect for task-type, where capacity was more limited in the distractor-absent than in the distractor-present task, *F*(1,42)  =  76.9, *p* <.001, *η_p_^2^*  = .65. Though the main effect of age-group did not reach significance, *F* <1, we found a significant interaction between age-group and task-type, *F*(1,42)  =  13.8, *p* <.001, *η_p_^2^*  = .25. Follow-up t-tests explicate this interaction. A significant age-related increase in capacity was only observed in the distractor-present task, *t*(42)  =  2.37, *p*  = .02 (marginally significant after a Bonferroni correction). In the distractor-absent task, this difference did not reach significance, *t*(42)  =  1.72, *p*  = .094 (ns, after a Bonferroni correction). Our LBA-based parametric approach, which follows below, complements and augments the C(*t*) and C*z* analyses.

### Parameter Estimates from Fitting the LBA Model

#### Model fit

**Figure 4:**
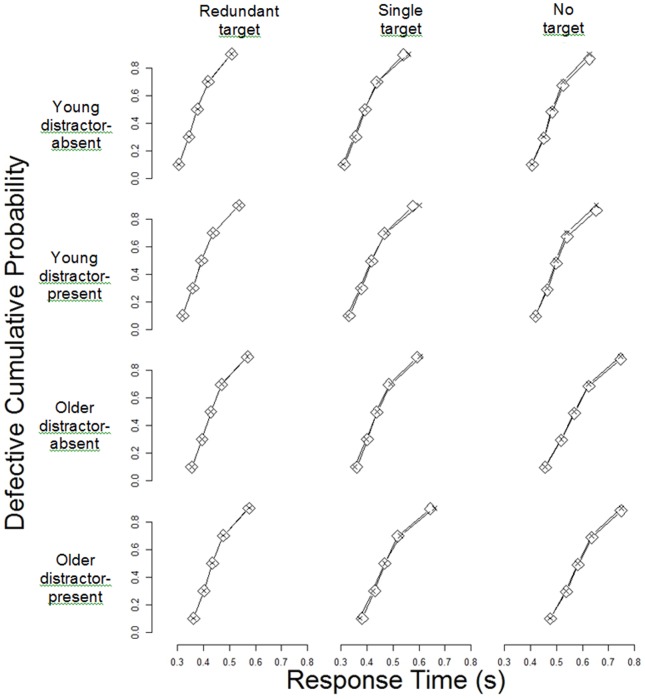
Cumulative Distribution Functions for correct responses from redundant-, single- and no-target trials, averaged over younger and older participants in distractor-present and distractor-absent tasks. Observed data are plotted using diamonds and model predictions using crosses.

As shown in Fig. 4, the model fit the data very well. In this figure we use cumulative distribution function plots to show the agreement between the predictions from the model and the observed data. Each set of points in Fig. 4 shows the defective cumulative probability (defective in the sense that they do not add up to 1, but instead sum to the probability that a correct or incorrect response was made) that the correct response is made as a function of response time (as summarized by the.1,.3,.5,.7 and .9 quantiles of the observed response time distribution). The height of the functions asymptote at the overall accuracy for each condition, and the extent to which the functions stretch across the horizontal axis represents the speed of responses. Fig. 4, therefore, shows simultaneously the speed and accuracy with which younger (rows 1-2) and older (rows 3-4) adults responded (correctly) in the redundant-, single- and no-target trials (columns 1-3, respectively) in the distractor-present (rows 1 and 3) and -absent (rows 2 and 4) tasks. Since the model fits the data well we can safely interpret the estimated parameters.

#### Model parameters: Accumulation rate

**Figure 5:**
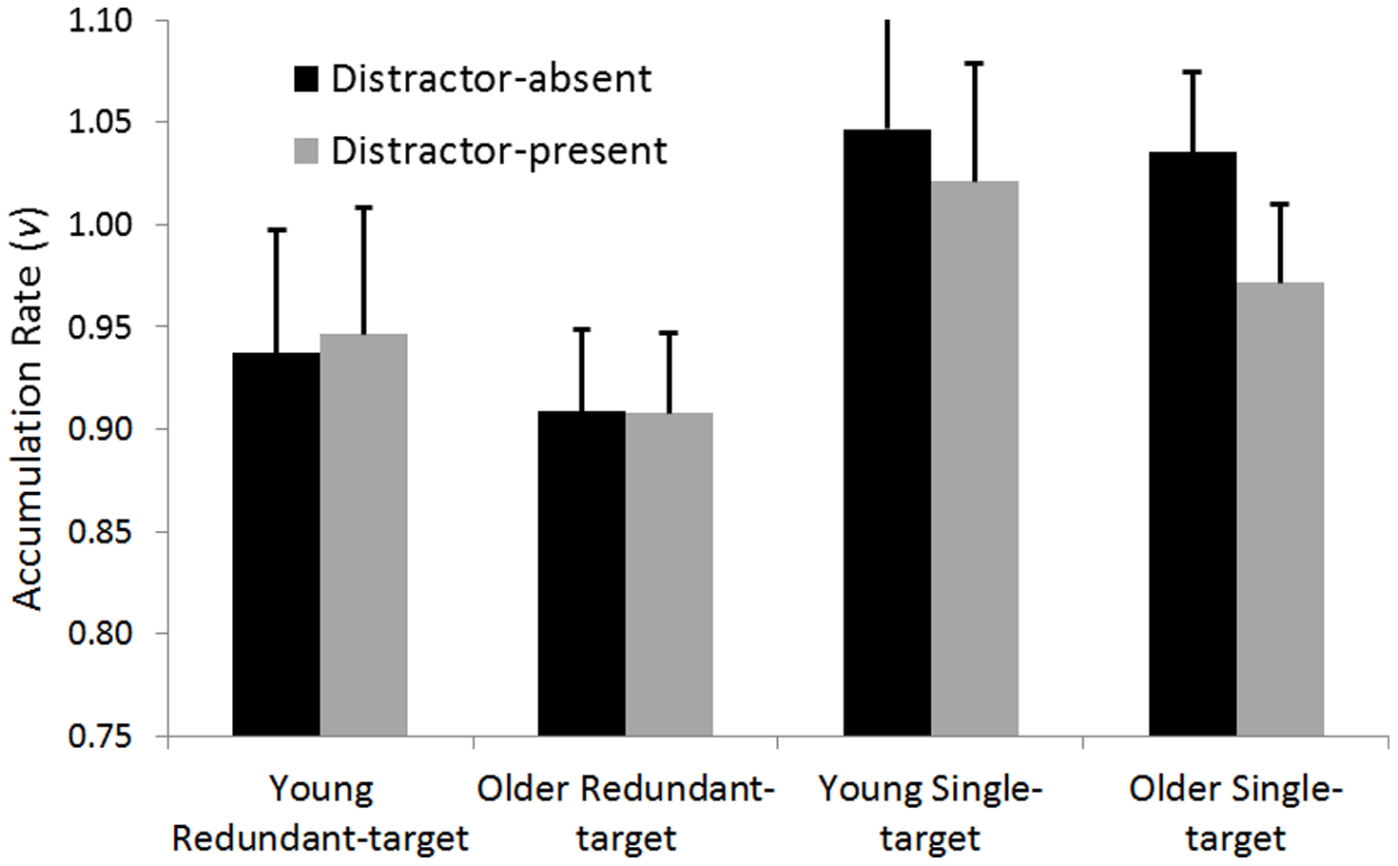
Accumulation rate parameters for the parametric model of the redundant target paradigm. The figure shows that in redundant-target trials, there are no effects for age and task-type. Yet in single-target trials, we observe a reduction in accumulation rates for older adults. Also note that accumulation rates for single-target trials are higher than for redundant-target trials, implying limited capacity.

Table 2 presents the best fitting parameters averaged across participants (but separated for task and age group). For the complete individual data, turn to S2 Table. Accumulation rates for target detection in single- and redundant-target trials in distractor-present and -absent tasks are plotted in Fig. 5. Note that, on average, the accumulation rates for each channel on redundant-target trials are slower than rates for single-target trials (means of.93 and 1.02 for redundant- and single-target trials, respectively), hinting that across age-groups and tasks capacity may be limited. Most revealing is the impact of distractors on accumulation rates: For redundant-target trials, the difference between distractor-present and -absent tasks appears small and negligible in both age-groups. Yet, for single-target trials, the impact of presenting a distractor in the display alongside the target appears to be much larger for older adults than for younger adults (the difference in rates between distractor-present and –absent tasks for older adults,.064, and for younger adults,.026).

**Table 2:** Average parameter values for the parametric model of the redundant target paradigm.

	Younger adults		Older adults	
	Distractor-absent	Distractor-present	Distractor-absent	Distractor-present
*A*	.10	.11	.10	.12
*b_Y_*	.374	.386	.384	.385
*b_N_*	.505	.515	.486	.498
*t_0_*	.110	.118	.148	.164
*v_RT_*	.937	.946	.909	.908
*v_ST_*	1.047	1.021	1.036	.972
*v_RT_/v_ST_*	.*887*	.*917*	.*873*	.*931*
*v_NT_*	Negative		Negative	
*v_∼T_*	Negative		Negative	
*v_∼NT_*	1.29		1.15	

*A* – start point variability; *b_Y_* and *b_N_* denote response thresholds for target-present and target-absent trials, respectively (Yes and No responses); *t_0_* – non-decision time; *v_RT_*, *v_ST_*, and *v_NT_* are accumulation rates for detection responses in redundant-target, single-target and no-target trials, respectively; *v_∼T_* and *v_∼NT_* stand for accumulation rates for rejection responses in target-present and target-absent trials, respectively. *Note:* v_NT_, v_∼T_ and *v_∼NT_* were averaged in each age-group across the two tasks (distractor-present and –absent); *v_NT_* and *v_∼T_* (representing mistakes: False alarms and miss, respectively) are very difficult to identify so their quantitative value is uninterpretable (they are large and negative). The estimated values for the parameters simply suggest that the corresponding accumulators very rarely win the race to threshold. Note that s, the between-trial variability in accumulation rate, was fixed at 0.25 for all accumulators.

To examine these effects, we replicate the omnibus ANOVA conducted for latencies with accumulation rates, using age-group (younger vs. older adults) as a between-participants factor, and task-type (distractor-present vs. -absent) and trial-type (single- vs. redundant-target trials), as within-participants factors. We note a main effect for trial-type, *F*(1,42)  =  482, *p* <.001, *η_p_^2^*  = .92, indicating larger rates for single-target trials; a main effect for task-type, *F*(1,42)  =  49.3, *p* <.001, *η_p_^2^*  = .54, reflecting slower accumulation rates in distractor-present tasks; and an interaction of the two factors, *F*(1,42)  =  55.7, *p* <.001, *η_p_^2^*  = .57, suggesting a smaller difference between the types of trials in the distractor-present task. Interestingly, age-group was not found to have a main effect on accumulation rates (*F* <1). Thus, there was no indication of an age-related generalized slowdown in the evidence-accumulation rates, across tasks and trials. Age-group, in contrast, was found to significantly interact with task-type, *F*(1,42)  =  17, *p* <.001, *η_p_^2^*  = .29, alongside a significant triple interaction of age-group, task-type and trial-type, *F*(1,42)  =  4.7, *p* <.05, *η_p_^2^*  = .1. Taking the two interactions together, the presence of distractors in the stimulus-set has a larger slowing impact on the accumulation rates of older adults, but this effect is larger for single-target trials.

Follow-up separate analyses of accumulation rates, in single-target trials and in redundant-target trials (using a Bonferroni correction for the possible 18 tests), untangles this interaction. In redundant-target trials, where no distractor can be present in the display, we found no effects for the type of task, the age-group, nor an interaction of the two (F <1, F <1, *F*(1,42)  =  2.7, *p*  = .1, respectively). This indicates that potential distraction has little impact on accumulation rates in redundant-target trials. Yet in the analysis of single-target trials, a significant interaction of age-group and task-type emerges, *F*(1,42)  =  12.5, *p*  = .001, *η_p_^2^*  = .23 (significant after a Bonferroni correction). So, the presence of a distractor in the display, alongside a target, slowed down accumulation rates, but only for older adults.

Parametric capacity estimates (*v_RT_*/*v_ST_*) follow the trend found in Townsend's non-parametric capacity. Mainly, distractors increase the capacity estimate for both age-groups, yet this increase is twice as large for older adults than for younger adults (average increase of 6.7% and 3.3% for older and younger adults, respectively, *F*(1,42)  =  4.89, *p* <.05). This pattern hints that the increase in capacity for older adults in the distractor-present task reflects a *decrease* in their ability to reject distractors (as evident by a decrease in accumulation rates in the single-target-single-distractor trial) rather than improved performance in redundancy (as age-group and task did not impact rates in redundant-target trials). Further evidence for the claim would come from analyzing the rate of accumulation for false alarms. Specifically, the rate of erroneously accumulating evidence for a target in its absence should increase when a distractor is present in the trial. Unfortunately, because participants were instructed to stress accuracy, mistakes were rare, and did not permit an analysis of accumulation rates.

**Figure 6:**
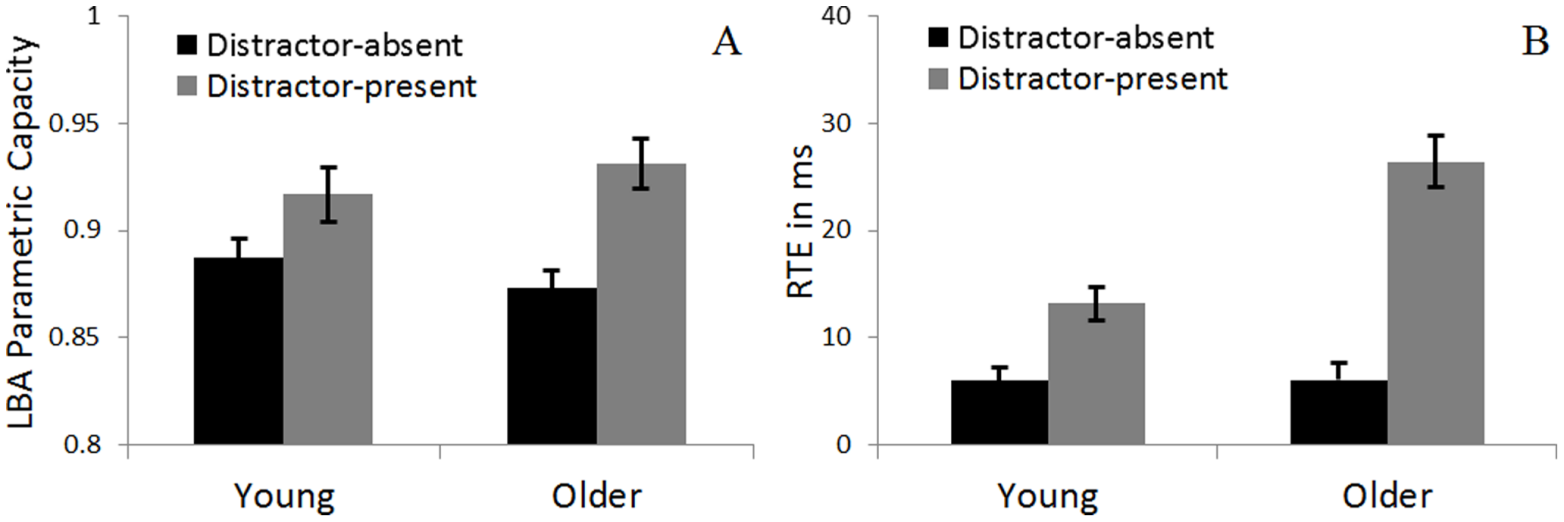
A comparison of LBA parametric capacity estimates *v_RT_*/*v_ST_*, Panel A and redundant target effect (RTE) in ms, RT(redundant-target) – RT(fastest single-target), Panel B. Comparing the two panels it is clear that the LBA parametric capacity follows the trend observed in the raw latency analysis. Mainly, adding a distractor to the task increases both measures, yet this increase is doubled for older adults.

Fig. 6 illustrates the similarity between model-based (LBA) parametric estimates of capacity and latency-based RTE, plotted in Panels A and B, respectively. Note that adding a distractor to the task increases both measures; and in both, the increase for older adults is approximately double the size of an increase for younger adults. This showcases the prowess of the LBA analysis in explaining age-related effects on RTE.

Can an age-related slowdown in speed of processing explain these effects? The results presented above provide no support for this option. We observed no main effect of aging on evidence accumulation rates, neither in the omnibus ANOVA, nor in the separate ANOVAs for single- and redundant-target trials (*F* <1 for all three). That is, older participants (with the exception of single-target-single-distractor trials) were able to extract and accumulate information from presented stimuli at the same rate as younger participants. Similarly, no age-related effects were found on the accumulation rates for correct rejections (no-target trials), *t*(42)  =  1.7, *p*  = .1. This result is, broadly speaking, consistent with the results that Ratcliff and colleagues have found in various applications of models of decision making to the aging literature [27], [31]–[33]. It is important to note that one of the main benefits of the parametric approach offered by the LBA is that parametric capacity coefficients have already taken into account the possibility of general age-related differences, such as generalized slowing. Thus, irrespective of changes in speed of processing, changes in parametric capacity coefficient can directly explain the specific age-related effects on RTE in the distractor-present task.

#### Model parameters: Non-decision time and response thresholds

Non-decision times, *t_0_*, represent sensory factors and motor factors that are known to deteriorate with aging. It is not surprising to find that older participants were estimated to be, on average, 42 ms slower than younger participants to complete the non-decision time components of response time, *F*(1,42)  =  8.8, *p*  = .005, *η_p_^2^*  = .17. Similarly, task-type had some impact (12 ms slowing) on non-decision time, *F*(1,42)  =  3.0, *p*  = .044 single-tail, *η_p_^2^*  = .07. The interaction of the two factors (task-type and age-group) failed to reach significance, *F* <1, even though, non-decision times increased in the distractor-present task by 16 ms for older adults, vs. only 8 ms for younger-adults.

Finally, the analysis of response thresholds shows a different pattern. Equivalent values were estimated for older and younger participants, (*b*  =  0.438 and 0.445, respectively, *F* <1), and for distractor-present and -absent task (*b*  =  0.437 and 0.446, respectively, *F* <1). The type of trial, however, was found to have a significant impact on response thresholds, *F*(1,42)  =  105, *p* <.001, *η_p_^2^*  = .61, with a higher threshold adopted in no-target trials. No significant 2-way or 3-way interactions were found for the three factors (age-trial, age-task, task-trial, or age-trial-task; in all *F*(1,42) ≤ 3, *p* ≥.1).

## Discussion

The redundant target task provides a convenient platform for testing how people cope with an increasing load of information. When asked to detect at least one target in a display, observers respond faster in double-target displays compared with single-target displays – RTE. Generally, studies have shown an age-related increase in RTE, but there remains a debate on the nature of the process underlying this increase: does it reflect improved processing of redundant signals with age, or does it reveal degraded performance in detection of single-targets? Do these differences stem from an age-related decrease in the efficiency of inhibition, or from a generalized slowing in the speed of processing? The results of the current study seem to support the inhibition of distractors account, where an increase in the capacity coefficient in aging reflects the difficulty that older adults experience in ignoring a distractor signal in single-target-single-distractor trials. Mainly, an age-related increase in RTE was only found when viewers had to actively inhibit (or suppress) the processing of a distractor present in a single-target trial. When no distractors were present, this age-related effect was erased. The age-related effect in distractor-present trials was stable even after controlling for changes in speed of processing. In a further analysis of the distribution of response latencies, employing both a non-parametric and a parametric approach, we observed the same results. An increase in the estimated processing capacity index for older adults was found to emanate from their specific difficulty in inhibiting a distractor in single-target-single-distractor trials, rather than from an improved ability to detect a target in redundant-target trials. No general age-related slowdown in the rate of evidence accumulation was found, further weakening the option that speed of processing could engender age-related effects on the RTE. The next sections examine each of the possible accounts in light of our results.

### Inhibition of Distractors

In the current study, involving detection of visual targets in two spatial positions, an age-related increase in RTE was observed only in a distractor-present task: where detection in double-target trials (*XX*) is compared to detection in single-target-single-distractor trials (*XO*). In distractor-absent task, where double-targets are compared with single-target-no-distractor trials (*X_*), RTEs were not impacted by age. These results were reinforced in an analysis of Townsend's non-parametric capacity coefficient, C(*t*), showing an age-related effect in the distractor-present task, but not in the distractor-absent one. This interaction of age-group and task was further indicated using C_Z_ statistics Together, these results appear to be in agreement with Hasher and Zacks' [20] theory on a decrease in the efficiency of inhibitory processes with aging. In the current task, this deficiency was marked by the inability of the elderly to ignore the irrelevant distractor, *O*, when looking for target letter *X*.

### Speed of Processing

Our latency analysis cannot be easily accommodated by a generalized slowing in speed of processing. Older adults were slower to respond than their younger counterparts, in both the distractor-present and -absent task, to the same extent (about 15%). Yet significant age-related effects on RTE were observed only in the distractor-present task. Moreover, the same pattern of results was documented after controlling for generalized cognitive slowing, in an analysis of log-transformed data (comparable to ratio effects). This suggests that the age-related increase in RTE in the distractor-present task was above and beyond any effect of generalized slowing. We note that the results can also reflect different age-related slowing rates for distractor-present and –absent tasks. Indeed, some researchers claim that different slowing functions with age relate to different task domains (for an example see [11]). Yet, in this case, both distractor -present and -absent tasks operate in the same domain (visual letter detection) – the only difference between the two is the additional distraction. Thus, it is unreasonable to assume that slowing will be governed by different slopes.

### LBA Analysis

This study presented a first adaptation to the literature on RTE and aging of a parametric model (LBA) for assessing workload capacity, decomposing the distribution of RT (and accuracy) into several latent variables. Age-related slowing in mean RT was found to derive mainly from non-decision time, *t_0_*, suggesting a change in response production factors, such as motor slowing. This change in *t_0_* could also be due to deterioration in sensory input factors — visual degradation. Following Wagenmakers' review [41], generalized slowing predicts an age-related steady slowing in the rate of the evidence accumulation, *v*. Yet, our analysis does not show a significant age-related difference in this factor, questioning the fit of this account for our data.

Evidence accumulation rates were used to derive a parametric capacity index (controlling for generalized slowing, response execution slowing, *t*_0_, and decision criterion, *b*) indicating a significantly larger age-related increase in capacity in the distractor-present task. Analysis of evidence accumulation rates suggests that this increase in the capacity index was because older adults were less efficient in processing single-target trials that were accompanied by distractors, while younger adults did not suffer as much. Thus, both parametric and non-parametric analyses coalesce to the same conclusion: *Relative* to their performance in single-target-single-distractor trials, older adults are more efficient in the processing of redundant-targets than younger adults. The performance of older adults was impaired in the distractor-present task and not improved with target-redundancy. This reduced efficiency is not related to general changes in speed of processing, but to difficulties in inhibiting distractors.

### Sensory Degradation

Finally, we wish to examine whether sensory changes (visual acuity) in aging, rather than cognitive changes (inhibition of distractors and speed of processing), may engender the noted increase in RTE. Notably, there are numerous studies that document visual sensory deterioration with age (see a review in [42]) and indicate its implication on various cognitive tasks [43], [44]. Visual sensory declines can directly affect performance on a variety of cognitive tasks, as the cognitive system has to deal with degraded information (see [45]; for a similar effect in a different modality see [46]). For example, Ben-David and Schneider [13], [47] have shown that age-related changes in a classic visual selective-attention task (color-word Stroop) can be partially explained by an age-related visual sensory degradation (see also [48], [49] for neuro-pathological examples). It is clear that visual degradation has an impact on visual search – when an older observer is searching for a target letter in a display, the system will receive degraded information on the identity (or the presence) of the letter, slowing down performance (see [50]).

Given a degraded input, older adults may confuse targets as distractors, slowing responses to single-targets in the distractor-present task but not to double-targets, resulting in a larger RTE. Conversely, in the distractor-absent task, a single-target trial is less likely to generate such confusion and RTE will not differ for younger and older adults (see a discussion in [17]). A somewhat similar view had been suggested by Kreuger and Allen [51], adopting the *internal noise model* [52], [53]. They postulated that the additional noise generated in the visual processing system of older adults may lead to such confusions of target and distractor. Yet these authors have rejected the *noise* hypothesis in later studies (e.g. [10]), as their attempt to mimic age-related visual degradation (by manipulating the luminance) did not inflate the RTE for younger adults.

Our LBA analysis does not provide a strong support for this account, either. On the one hand, the main reason for slowdown in aging in the current study appears to be a decrease in non-decision time, *t_0_*, which may relate to such sensory declines (e.g. [41]). Yet, if sensory degradation engenders the effect, one could expect an increase in *t_0_* specific for older adults in the distractor-present task (where an age-related change in RTE occurs), and not in the distractor-absent task (where it does not). We did not observe this pattern, but rather the increase in *t_0_* was general across all conditions. Likewise, older adults did not make more false detections or miss responses in the distractor-present task than in the distractor-absent one, as expected if sensory degradation decreases the discriminability between target and distractor. Clearly, future research is needed to further test this account.

### Future Directions

The current study employed a simple (yet extremely informative) factorial design, where up to two items could be displayed simultaneously. It is possible that differences in workload capacity between younger and older adults are even more prominent with more than two items to process. Future studies may focus on parametric manipulation of the number of items (distractors and targets) to increase the load. Fortunately, the necessary mathematical models for calculating C(*t*) for n>2 items are trivial and the formulae are readily available (e.g., [40]).

It is also possible that the designation of items as targets and distractors (and not only the number of items) might have had an impact on our results. However, existing literature suggests otherwise. For example, Eidels et al. [7] used a design akin to ours to calculate C(*t*) for the processing of Stroop color-word stimuli and found no effect of target assignment. In their study (Exp. 2), a color word (either *RED* or *GREEN*) would appear, printed in either red or green font color. Participants were asked to detect the presence of *redness*, i.e., to respond affirmatively if they detect the word *RED*, the color red, or both, and C(*t*) was calculated from RTs on the single- (*RED* printed in green, *GREEN* in red) and double-target (*RED* in red) trials. In a subsequent experiment (Exp. 3), exactly the same stimuli were used, except that target assignment was different and participants were asked to detect the target word *RED* and the target color green. Despite this semantically consequential difference, the capacity results were similar across the two experiments. This suggests that it should matter little whether the target was *X* or *O*, red or green. Of course, certain scenarios could exist in which the processing of the target is extremely difficult, whereas the processing of distractors is very easy (or vice versa). The prevalent use of *X* and *O* in redundant target studies (see Table 1 in [5]) suggests it is not likely. Future studies may further investigate target and distractor nature and designation.

### Conclusions

In analyses of both RT means and distributions, our results appear to support an inhibition of distractors source for the age-related increase in redundancy gains and measures of workload capacity. The data do not support a change in speed of processing as the main source for the increase in RTE. This conclusion is supported by converging evidence from all three measures tested. Finally, our results showcase the prowess of redundancy to significantly improve performance for older adults, in cases where noise signals might be present. This reinforces the notion that by minimizing the presence of distractors in the environment, we can improve the performance of older adults. However, given that in many cases distractors cannot be avoided, it is advisable to consider using redundancy of signals, when designing displays for older adults.

## Supporting Information

S1 Appendix **The Capacity Coefficient, C(t).**
(DOCX)

S1 Table **A summary of response latencies (in ms) for each participant, across conditions.**
(DOCX)

S2 Table **A summary of parameter values for the parametric model of the redundant target paradigm, for each participant across conditions.**
(DOCX)
