# Supplementary material for: Effects of Aging and Distractors on Detection of Redundant Visual Targets and Capacity: Do Older Adults Integrate Visual Targets Differently than Younger Adults?
Source: PLoS One. 2014 Dec 12;9(12):e113551. doi: 10.1371/journal.pone.0113551 (PMC4264737; doi:10.1371/journal.pone.0113551)
Supplement: S1 Appendix — The Capacity Coefficient, C(t). (DOCX) [file pone.0113551.s001.docx]

Appendix: The Capacity Coefficient, C(*t*)

*Workload capacity* refers to the fundamental ability of a system to deal with heavier task duties ([54,55]; and more recently [4]). In the context of the current study, it may refer to the efficiency of processing item A when another item, B, had been added to the display. Processing speed in the latter condition, essentially the double-target condition (AB), can be compared to a single-target condition, where A is presented in isolation. Indeed, Townsend and Nozawa [6] proposed a measure of performance on double-target trials -- the *capacity coefficient* -- which is the ratio of the integrated hazard function of the double-target condition to the sum of the integrated hazard functions of the single-target conditions:

. (A1)

Here, we define the survivor function as the complement of the cumulative distribution function, S(*t*)=1-F(*t*); the hazard function as the probability density function divided by the survivor function, h(*t*)=f(*t*)/S(*t*); and the integrated hazard function, H(*t*) as the integral of the hazard function from zero to *t*. Townsend and others (e.g. [56]) showed that H(*t*) = - log[S(*t*)], which allows easy estimation of H(*t*) and consequently C(*t*) from empirical data. The subscripts OR indicate that this measure is adequate for an OR task, where participants are asked to detect a target on one position *or* another. Townsend and Wenger [56] developed a comparable measure for the AND task, where participants respond affirmatively only if targets appear on both positions, but we make no use of this index in the current study.

Processing efficiency of the system, when measured by C_OR_(*t*), is compared against the standard parallel model (i.e., a parallel model with unlimited capacity, and with stochastically independent channels) as a benchmark. If C_OR_(*t*) = 1 for all values of t, performance is identical to that of the standard parallel model. That is, C_OR_(*t*) values of 1 imply that the system has unlimited *capacity*, in the sense that processing in a given channel is not affected by the increase in work load due to the increase in the number of targets. Any given channel in that case has the same processing rate whether a target is presented to the other channel or not. C_OR_(*t*) values that are below 1 imply that capacity is limited, such that increasing the processing load by increasing the number of targets on the display takes a toll on performance. In that case, the processing in a given channel is slowed down by the simultaneous operation of another channel. Finally, if C_OR_(*t*) > 1 then the system is said to have super-capacity, where processing efficiency of individual channels increases with increased work load.

Some researchers employ a different measure to assess the efficiency of processing multiple targets. Miller [2,57] introduced the *race model inequality*, which puts an upper bound on performance if the system is an independent race. Violations of this bound were taken to imply coactivation, a special case of parallel processing where activation from several channels is pooled together to a common conduit, allowing responses with double-target to be particularly fast. Several studies employed Miller’s race-model inequality to explore differences in multi-sensory integration across younger and older adults (e.g. [58]). Notably, Townsend and Eidels [4] showed that the race model inequality can be expressed mathematically using survivor functions in such way that allows it to be plotted against the C(*t*) function. Under this interpretation, the race model inequality can be viewed as a (conservative) bound for super-capacity rather than a test for coactivation. The formal proofs are beyond the current scope but are available in their paper.

References:

54. Kahneman D (1973) Attention and effort. Englewood Cliffs, NJ: Prentice Hall.

55. Townsend JT, Ashby FG (1983) The stochastic modeling of elementary psychological processes. Cambridge, UK: Cambridge University Press.

56. Townsend JT, Wenger MJ (2004) A theory of interactive parallel processing: New capacity measures and predictions for a response time inequality series. Psychological Review, 111(4): 1003-1035 doi: 10.1037/0033-295X.111.4.1003

57. Miller J (1978) Multidimensional same-different judgments: Evidence against independent comparisons of dimensions. J Exp Psychol Hum Percept Perform 4(3): 411-422 doi: [10.1037/0096-1523.4.3.411](http://psycnet.apa.org/doi/10.1037/0096-1523.4.3.411)

58. Laurienti P, Burdette JH, Maldjian JA, Wallace MT (2006) Enhanced multisensory in older adults. Neurobiol Aging 27(8): 1155–1163. doi: 10.1016/j.neurobiolaging.2005.05.024
