# Supplementary material for: Effects of Aging and Distractors on Detection of Redundant Visual Targets and Capacity: Do Older Adults Integrate Visual Targets Differently than Younger Adults?
Source: PLoS One. 2014 Dec 12;9(12):e113551. doi: 10.1371/journal.pone.0113551 (PMC4264737; doi:10.1371/journal.pone.0113551)
Supplement: S1 Table — A summary of response latencies (in ms) for each participant, across conditions. (DOCX) [file pone.0113551.s002.docx]

|  |  | Distractor-absent | | | | | | Distractor-present | | | | | |
| --- | --- | --- | --- | --- | --- | --- | --- | --- | --- | --- | --- | --- | --- |
|  |  | Redundant-target | Faster single-target | No-target | Average RT | RTE | RTE log-transform | Redundant-target | Faster single-target | No-target | Average RT | RTE | RTE log-transform |
| Younger adults | 1 | 406 | 410 | 512 | 434 | 4 | .0052 | 424 | 440 | 557 | 467 | 17 | 0.0178 |
|  | 2 | 458 | 462 | 549 | 496 | 5 | .0062 | 494 | 488 | 573 | 519 | -7 | -0.0028 |
|  | 3 | 350 | 347 | 418 | 374 | -3 | -.0031 | 338 | 360 | 419 | 371 | 22 | 0.0266 |
|  | 4 | 340 | 344 | 438 | 367 | 4 | .0048 | 352 | 381 | 457 | 394 | 29 | 0.0327 |
|  | 5 | 377 | 388 | 505 | 418 | 11 | .0113 | 428 | 450 | 540 | 472 | 22 | 0.0202 |
|  | 6 | 323 | 336 | 410 | 356 | 13 | .0166 | 320 | 336 | 408 | 354 | 16 | 0.0199 |
|  | 7 | 351 | 361 | 469 | 393 | 9 | .0115 | 391 | 393 | 489 | 431 | 2 | 0.005 |
|  | 8 | 371 | 379 | 466 | 401 | 7 | .0073 | 378 | 391 | 473 | 414 | 13 | 0.0141 |
|  | 9 | 417 | 417 | 506 | 443 | 0 | .0012 | 427 | 444 | 525 | 462 | 18 | 0.0177 |
|  | 10 | 327 | 327 | 421 | 357 | 0 | -.0006 | 359 | 376 | 443 | 390 | 18 | 0.0218 |
|  | 11 | 388 | 398 | 473 | 424 | 10 | .0110 | 364 | 368 | 459 | 396 | 5 | 0.0056 |
|  | 12 | 374 | 372 | 450 | 396 | -2 | -.0004 | 392 | 391 | 468 | 416 | -1 | 0 |
|  | 13 | 420 | 423 | 507 | 444 | 3 | .0046 | 448 | 441 | 527 | 467 | -7 | -0.0056 |
|  | 14 | 334 | 345 | 462 | 373 | 10 | .0126 | 343 | 362 | 462 | 381 | 19 | 0.0233 |
|  | 15 | 362 | 366 | 435 | 385 | 4 | .0050 | 361 | 371 | 441 | 388 | 10 | 0.0113 |
|  | 16 | 486 | 499 | 601 | 520 | 13 | .0109 | 527 | 533 | 616 | 555 | 5 | 0.0039 |
|  | 17 | 417 | 427 | 510 | 447 | 10 | .0103 | 409 | 434 | 495 | 449 | 24 | 0.0232 |
|  | 18 | 483 | 504 | 669 | 537 | 21 | .0189 | 523 | 534 | 709 | 574 | 12 | 0.0111 |
|  | 19 | 348 | 355 | 460 | 382 | 7 | .0085 | 400 | 418 | 495 | 438 | 18 | 0.0196 |
|  | 20 | 474 | 473 | 571 | 500 | -1 | -.0005 | 474 | 497 | 587 | 514 | 22 | 0.0201 |
|  | 21 | 356 | 361 | 460 | 391 | 5 | .0064 | 391 | 410 | 467 | 431 | 19 | 0.0196 |
|  | 22 | 423 | 425 | 512 | 453 | 2 | .0019 | 396 | 411 | 498 | 439 | 15 | 0.0165 |
| Older adults | 1 | 397 | 405 | 486 | 430 | 8 | .0069 | 441 | 464 | 570 | 499 | 24 | 0.022 |
|  | 2 | 512 | 511 | 559 | 534 | -1 | -.0010 | 513 | 531 | 583 | 544 | 18 | 0.0155 |
|  | 3 | 530 | 551 | 599 | 561 | 21 | .0146 | 534 | 565 | 610 | 582 | 31 | 0.0228 |
|  | 4 | 433 | 435 | 560 | 467 | 3 | .0041 | 459 | 489 | 617 | 513 | 30 | 0.03 |
|  | 5 | 400 | 406 | 502 | 430 | 6 | .0058 | 423 | 463 | 532 | 471 | 41 | 0.039 |
|  | 6 | 403 | 410 | 500 | 432 | 6 | .0063 | 414 | 425 | 522 | 453 | 11 | 0.0114 |
|  | 7 | 402 | 398 | 493 | 423 | -4 | -.0028 | 397 | 417 | 522 | 440 | 20 | 0.02 |
|  | 8 | 416 | 421 | 541 | 455 | 5 | .0068 | 425 | 451 | 548 | 473 | 26 | 0.0259 |
|  | 9 | 394 | 400 | 484 | 421 | 6 | .0051 | 410 | 433 | 512 | 454 | 24 | 0.0225 |
|  | 10 | 497 | 513 | 601 | 532 | 15 | .0119 | 494 | 520 | 622 | 539 | 26 | 0.0207 |
|  | 11 | 377 | 378 | 441 | 400 | 1 | .0018 | 397 | 443 | 487 | 443 | 47 | 0.0469 |
|  | 12 | 385 | 385 | 543 | 432 | 0 | .0032 | 472 | 506 | 606 | 536 | 34 | 0.0296 |
|  | 13 | 409 | 411 | 444 | 421 | 2 | .0028 | 433 | 451 | 513 | 475 | 17 | 0.0167 |
|  | 14 | 437 | 457 | 530 | 471 | 19 | .0133 | 496 | 543 | 567 | 537 | 47 | 0.0378 |
|  | 15 | 407 | 405 | 702 | 481 | -2 | -.0019 | 447 | 454 | 649 | 501 | 7 | 0.0074 |
|  | 16 | 524 | 520 | 627 | 550 | -3 | -.0023 | 361 | 371 | 441 | 388 | 10 | 0.0113 |
|  | 17 | 495 | 493 | 713 | 552 | -2 | -.0013 | 489 | 523 | 775 | 579 | 34 | 0.0292 |
|  | 18 | 450 | 454 | 841 | 544 | 4 | .0047 | 470 | 484 | 871 | 579 | 14 | 0.0119 |
|  | 19 | 519 | 533 | 561 | 538 | 13 | .0125 | 508 | 543 | 560 | 546 | 35 | 0.0254 |
|  | 20 | 547 | 559 | 675 | 582 | 12 | .0084 | 558 | 574 | 661 | 591 | 16 | 0.013 |
|  | 21 | 434 | 450 | 739 | 522 | 16 | .0108 | 449 | 485 | 747 | 546 | 36 | 0.0356 |
|  | 22 | 425 | 434 | 554 | 465 | 9 | .0089 | 405 | 439 | 546 | 461 | 34 | 0.0336 |
