# Supplementary material for: Effects of Aging and Distractors on Detection of Redundant Visual Targets and Capacity: Do Older Adults Integrate Visual Targets Differently than Younger Adults?
Source: PLoS One. 2014 Dec 12;9(12):e113551. doi: 10.1371/journal.pone.0113551 (PMC4264737; doi:10.1371/journal.pone.0113551)
Supplement: S2 Table — A summary of parameter values for the parametric model of the redundant target paradigm, for each participant across conditions. (DOCX) [file pone.0113551.s003.docx]

|  |  | Distractor-absent | | | | | | Distractor-present | | | | | |
| --- | --- | --- | --- | --- | --- | --- | --- | --- | --- | --- | --- | --- | --- |
|  |  | *b_Y_* | *b_N_* | *t_0_* | *v_RT_* | *v_ST_* | *v_RT_/v_ST_* | *b_Y_* | *b_N_* | *t_0_* | *v_RT_* | *v_ST_* | *v_RT_/v_ST_* |
| Younger adults | 1 | 0.27 | 0.3 | 0.17 | 0.69 | 0.83 | 0.82 | 0.26 | 0.32 | 0.18 | 0.7 | 0.77 | 0.92 |
|  | 2 | 0.47 | 0.59 | 0.11 | 0.87 | 0.96 | 0.9 | 0.49 | 0.61 | 0.12 | 0.86 | 1 | 0.86 |
|  | 3 | 0.31 | 0.46 | 0.08 | 0.94 | 1.04 | 0.9 | 0.34 | 0.48 | 0.11 | 0.94 | 1.02 | 0.93 |
|  | 4 | 0.37 | 0.58 | 0.06 | 1.02 | 1.14 | 0.9 | 0.42 | 0.62 | 0.07 | 1.02 | 1.08 | 0.95 |
|  | 5 | 0.2 | 0.41 | 0.21 | 0.81 | 0.9 | 0.9 | 0.3 | 0.54 | 0.16 | 0.82 | 0.88 | 0.94 |
|  | 6 | 0.72 | 0.99 | 0.03 | 1.65 | 1.73 | 0.95 | 0.64 | 0.93 | 0.05 | 1.67 | 1.7 | 0.98 |
|  | 7 | 0.26 | 0.37 | 0.18 | 0.85 | 0.93 | 0.91 | 0.33 | 0.41 | 0.17 | 0.82 | 0.92 | 0.89 |
|  | 8 | 0.47 | 0.6 | 0.06 | 1.2 | 1.31 | 0.92 | 0.42 | 0.55 | 0.09 | 1.24 | 1.26 | 0.98 |
|  | 9 | 0.44 | 0.56 | 0.09 | 1 | 1.12 | 0.89 | 0.52 | 0.66 | 0.04 | 1.01 | 1.09 | 0.93 |
|  | 10 | 0.24 | 0.39 | 0.1 | 0.87 | 0.97 | 0.9 | 0.25 | 0.38 | 0.13 | 0.88 | 0.94 | 0.94 |
|  | 11 | 0.49 | 0.54 | 0.1 | 1.1 | 1.18 | 0.93 | 0.4 | 0.48 | 0.08 | 1.14 | 1.19 | 0.96 |
|  | 12 | 0.37 | 0.56 | 0.12 | 1 | 1.14 | 0.87 | 0.37 | 0.56 | 0.14 | 0.99 | 1.12 | 0.89 |
|  | 13 | 0.4 | 0.5 | 0.03 | 0.83 | 0.95 | 0.87 | 0.46 | 0.54 | 0.06 | 0.84 | 0.97 | 0.87 |
|  | 14 | 0.25 | 0.46 | 0.15 | 0.88 | 0.99 | 0.89 | 0.22 | 0.41 | 0.19 | 0.87 | 0.95 | 0.91 |
|  | 15 | 0.53 | 0.59 | 0.09 | 1.61 | 1.71 | 0.94 | 0.56 | 0.64 | 0.1 | 1.63 | 1.69 | 0.97 |
|  | 16 | 0.27 | 0.36 | 0.19 | 0.48 | 0.63 | 0.77 | 0.33 | 0.4 | 0.14 | 0.48 | 0.61 | 0.78 |
|  | 17 | 0.46 | 0.58 | 0.1 | 0.97 | 1.09 | 0.89 | 0.39 | 0.51 | 0.13 | 0.99 | 1.02 | 0.97 |
|  | 18 | 0.3 | 0.49 | 0.1 | 0.49 | 0.59 | 0.83 | 0.34 | 0.52 | 0.13 | 0.47 | 0.62 | 0.77 |
|  | 19 | 0.28 | 0.43 | 0.12 | 0.9 | 1 | 0.9 | 0.35 | 0.48 | 0.12 | 0.9 | 0.97 | 0.93 |
|  | 20 | 0.49 | 0.56 | 0.06 | 0.72 | 0.87 | 0.83 | 0.4 | 0.48 | 0.11 | 0.74 | 0.83 | 0.89 |
|  | 21 | 0.28 | 0.41 | 0.11 | 0.75 | 0.85 | 0.88 | 0.34 | 0.43 | 0.12 | 0.76 | 0.82 | 0.93 |
|  | 22 | 0.35 | 0.4 | 0.17 | 1.01 | 1.1 | 0.91 | 0.35 | 0.4 | 0.16 | 1.05 | 1.04 | 1.01 |
| Older adults | 1 | 0.55 | 0.64 | 0.04 | 1.32 | 1.4 | 0.94 | 0.55 | 0.67 | 0.1 | 1.29 | 1.35 | 0.95 |
|  | 2 | 0.39 | 0.49 | 0.23 | 0.96 | 1.06 | 0.9 | 0.38 | 0.52 | 0.24 | 0.95 | 1.04 | 0.92 |
|  | 3 | 0.56 | 0.58 | 0.1 | 0.99 | 1.09 | 0.91 | 0.46 | 0.5 | 0.16 | 0.96 | 1.01 | 0.95 |
|  | 4 | 0.41 | 0.52 | 0.13 | 0.84 | 0.98 | 0.85 | 0.46 | 0.59 | 0.06 | 0.82 | 0.91 | 0.9 |
|  | 5 | 0.42 | 0.48 | 0.13 | 1.11 | 1.24 | 0.89 | 0.45 | 0.51 | 0.12 | 1.13 | 1.13 | 1 |
|  | 6 | 0.31 | 0.48 | 0.18 | 0.97 | 1.1 | 0.89 | 0.4 | 0.6 | 0.14 | 0.97 | 1.06 | 0.91 |
|  | 7 | 0.36 | 0.43 | 0.22 | 0.93 | 1.15 | 0.8 | 0.36 | 0.51 | 0.17 | 0.93 | 1.03 | 0.9 |
|  | 8 | 0.33 | 0.37 | 0.15 | 0.92 | 1.02 | 0.9 | 0.31 | 0.36 | 0.21 | 0.95 | 0.96 | 0.99 |
|  | 9 | 0.28 | 0.34 | 0.25 | 1.03 | 1.2 | 0.86 | 0.34 | 0.43 | 0.21 | 0.99 | 1.06 | 0.93 |
|  | 10 | 0.71 | 0.95 | 0.05 | 1.12 | 1.25 | 0.9 | 0.66 | 0.89 | 0.14 | 1.14 | 1.26 | 0.9 |
|  | 11 | 0.29 | 0.32 | 0.17 | 0.89 | 1.01 | 0.88 | 0.3 | 0.35 | 0.22 | 0.92 | 0.91 | 1.01 |
|  | 12 | 0.47 | 0.58 | 0.09 | 1.05 | 1.17 | 0.9 | 0.62 | 0.67 | 0.13 | 1.07 | 1.1 | 0.97 |
|  | 13 | 0.31 | 0.29 | 0.2 | 1.01 | 1.15 | 0.88 | 0.37 | 0.39 | 0.15 | 1.01 | 1.03 | 0.98 |
|  | 14 | 0.28 | 0.31 | 0.16 | 0.7 | 0.82 | 0.86 | 0.27 | 0.29 | 0.22 | 0.69 | 0.74 | 0.94 |
|  | 15 | 0.26 | 0.38 | 0.13 | 0.74 | 0.86 | 0.85 | 0.29 | 0.35 | 0.18 | 0.71 | 0.88 | 0.81 |
|  | 16 | 0.52 | 0.59 | 0.1 | 1.03 | 1.16 | 0.89 | 0.28 | 0.34 | 0.19 | 1.02 | 1.13 | 0.91 |
|  | 17 | 0.41 | 0.57 | 0.03 | 0.72 | 0.86 | 0.84 | 0.44 | 0.65 | 0.07 | 0.72 | 0.81 | 0.88 |
|  | 18 | 0.23 | 0.56 | 0.22 | 0.7 | 0.84 | 0.84 | 0.28 | 0.64 | 0.14 | 0.7 | 0.77 | 0.9 |
|  | 19 | 0.48 | 0.52 | 0.16 | 0.91 | 1.03 | 0.88 | 0.49 | 0.53 | 0.16 | 0.94 | 0.97 | 0.96 |
|  | 20 | 0.26 | 0.3 | 0.18 | 0.48 | 0.61 | 0.78 | 0.26 | 0.29 | 0.26 | 0.48 | 0.61 | 0.8 |
|  | 21 | 0.28 | 0.56 | 0.2 | 0.66 | 0.79 | 0.84 | 0.24 | 0.54 | 0.14 | 0.68 | 0.69 | 0.99 |
|  | 22 | 0.36 | 0.43 | 0.14 | 0.92 | 1.02 | 0.9 | 0.28 | 0.37 | 0.2 | 0.92 | 0.94 | 0.98 |
